# Association between the use of fitness trackers, perfectionism, motivation and anxiety in athletes

**DOI:** 10.3389/fpsyg.2026.1805652

**Published:** 2026-04-16

**Authors:** Irene Huguet-López, Silvia Trujillo Barberá, Clara López-Mora

**Affiliations:** 1 Departamento de Psicología, Universidad Europea de Valencia, Valencia, Spain; 2 Facultad de Ciencias de la Salud, Universidad Europea de Valencia, Valencia, Spain

**Keywords:** fitness trackers, perfectionism, motivation, anxiety, athletes

## Abstract

**Introduction:**

In recent years, the use of fitness trackers in sports practice has increased. However, despite their growing popularity, there is little empirical evidence regarding their psychological impact on non-professional athletes, particularly in relation to variables such as motivation, anxiety, and perfectionism. Therefore, the aim of this study was to explore possible differences and associations in these psychological variables based on the use of fitness trackers.

**Methods:**

A total of 120 athletes participated and completed three questionnaires: the Sport-MPS-2, the SMS, and the SAS-2. A comparative cross-sectional study of independent groups with non-probabilistic sampling was carried out.

**Results:**

Significant differences were observed in the variables of personal standards (U = 861, *p* = 0.002) and concern about mistakes (U = 873, *p* < 0.001) and concentration disruption (0 = 1.097, *p* = 0.034).

**Discussion:**

These findings suggest an association between the use of fitness trackers and personal standards and concern about mistakes. The association with motivation-related variables depends mainly on the reasons for their use rather than the device itself. Feedback from fitness trackers could act as a cognitive anchor, allowing greater attention to the task at hand. Negative emotional and cognitive associations may be influenced by other variables. The need for further examination of these relationships through larger studies and discipline-specific research is evident.

## 1 Introduction

In recent years, there has been a notable increase in the use of fitness trackers, also known as wearable electronic devices (Saxena and Singh, 2025). This boom is reflected in economic terms: the global market for fitness trackers was valued at $53.94 billion in 2023 and is projected to reach $290.85 billion by 2032 (Fortune Business Insights, 2024). These devices integrate biometric sensors into everyday objects such as watches, bracelets, or sportswear and allow users to measure and record data related to physical activity (heart rate, speed, distance traveled, or intensity of effort). The information collected can be viewed in real time and shared with others through mobile applications (Camas-Ramírez, 2017).

Although the literature highlights the psychological benefits associated with the use of fitness trackers (Choudhury and Asan, 2021; Karapanos et al., 2016; Ryan et al., 2019), possible adverse associations have also been noted, such as feelings of intrusion or loss of autonomy (Xue, 2019). Both gray literature (Cooray and Duus, 2015; Davies, 2018) and scientific studies (Chen et al., 2024; Fleer, 2024) have warned of a possible “dark side” to these devices. The use of fitness trackers involves constant interaction with data, external feedback, and social elements that are related to the user’s subjective experience (Etkin, 2016; Goodyear et al., 2019; Zahrt et al., 2023). These dynamics can trigger psychological processes that have not yet been sufficiently explored in the context of amateur sports. Therefore, the objective of this study is to explore the associations and differences between psychological variables such as anxiety, perfectionism, and motivation according to the use of fitness trackers.

Perfectionism is a personality trait characterized by a tendency to set unrealistic standards and be overly critical. People with this trait have difficulty adapting due to their high and unrealistic standards and the effort required to achieve them. In addition, they pay selective attention to failures, generalizing them, and conducting strict self-evaluations. They tend to think in terms of all or nothing, leading to the belief that there is only total success or failure (Chemisquy, 2018; Frost et al., 1990; Hewitt and Flett, 1991; Nguyen and Morris, 2024). Over the last three decades, two models of perfectionism have been developed. One of them, proposed by Frost et al. (1990), comprises six aspects: personal standards (setting high standards of evaluation), concern about mistakes (negative reactions to mistakes), doubts about actions (tendency to doubt one’s own ability), parental expectations (belief that parents set very high standards), parental criticism (belief that parents are overly critical), and organization (importance attributed to order). On the other hand, Hewitt and Flett's (1991) model proposes three dimensions: self-oriented perfectionism (perfectionist behaviors directed at oneself), other-oriented perfectionism (perfectionist behaviors directed at others), and socially prescribed perfectionism (behaviors directed at achieving the norms and expectations prescribed by other people). Although both models are related, they do not completely overlap. The results of the factor analysis of the nine subscales indicated a two-factor solution (Frost et al., 1993; Parks et al., 2021; Robinson et al., 2022): perfectionist concerns (including the subscales concern about mistakes, parental expectations, parental criticism, doubts about actions, and socially prescribed perfectionism), which correlated positively with depression and negative affectivity. These are considered the most dysfunctional facet of this construct, as they have been observed to reduce commitment and involvement in tasks (Jara-Moreno et al., 2020). On the other hand, perfectionist efforts (including the subscales of personal standards, self-oriented perfectionism, organization, and other-oriented perfectionism) correlated positively with positive affectivity.

Perfectionism has been conceptualized as a transdiagnostic vulnerability factor common to various psychological disorders, such as eating disorders and obsessive-compulsive disorder (Gerdan and Salcioglu, 2025). Likewise, perfectionist concerns have been consistently linked to greater vulnerability to problems such as anxiety and depression (Gil-Caselles et al., 2023; Simon et al., 2025; Tóth et al., 2022). In the athletic population, both socially prescribed and self-oriented perfectionism have been found to be indirectly associated with lower levels of self-esteem and self-competence using maladaptive emotional regulation strategies, such as self-blame and catastrophizing (Minichiello et al., 2024).

Anxiety is an emotional state characterized by the activation of multiple components: cognitive, physiological, emotional, and behavioral. It usually manifests as nervousness, fear, or worry, and can arise in response to stimuli that the individual perceives as threatening, even if these are not objectively present (Martens et al., 1990). This emotional state is particularly activated when there is a discrepancy between the demands of the context (or self-imposed demands) and the perceived ability to respond adequately to those demands (Lazarus and Folkman, 1984; Martens et al., 1990). In the sporting context, this type of activation can be intensified by performance pressure, public exposure, or self-imposed demands (González-Hernández et al., 2021). According to the multidimensional theory of anxiety (Martens et al., 1990), this construct consists of two dimensions: cognitive anxiety and somatic anxiety. The former refers to the mental processes involved in the perception of threat, such as intrusive thoughts, worries, difficulty concentrating, or negative anticipation (Martens et al., 1990). Specifically, worry refers to concern about the potentially negative consequences associated with poor performance, while concentration disruption is associated with the athlete’s difficulty in focusing on the key aspects of the task at hand, which prevents clear thinking during the sporting situation (Grossbard et al., 2009). The latter refers to associated physiological responses, such as muscle tension, increased heart rate, stomach discomfort, or tremors (Martens et al., 1990). Both dimensions can alter the athlete’s psychological and physical functioning, affecting their ability to maintain attention, regulate their emotions, or perform effectively (Haase, 2021).

Anxiety does not affect all individuals in the same way, as its intensity and expression depend on factors such as context, previous experience, or the athlete’s psychological profile (Haase, 2021; Jara-Moreno et al., 2020). A study conducted by Tóth et al., 2022 concluded that irrational beliefs were related to cognitive and somatic anxiety through the variable of perfectionism. Both the more functional and dysfunctional facets were found to be partial mediators of irrational beliefs about anxiety. In a systematic review conducted by Chen et al., 2024 analyzing the negative effects of these devices, it was concluded that 42 of the articles analyzed (64.4%) reported some type of negative emotion, the most common being guilt, anxiety, and frustration.

Motivation in sport has been studied from various theoretical approaches. One of the most prominent is self-determination theory (SDT) (Ryan and Deci, 1985; Ryan and Deci, 2020). According to this theory, human beings have an innate tendency toward psychological growth, seeking to integrate their experiences and behaviors in a way that is consistent with their values and interests.

Within SDT, the theory of organic integration establishes a motivational continuum that encompasses different degrees of self-determination of behavior. This taxonomy encompasses three types of motivation according to their position on the continuum: extrinsic motivation, intrinsic motivation, and demotivation. Intrinsic motivation refers to the need to explore the environment, curiosity, and the pleasure experienced when performing an activity. Within intrinsic motivation, there is intrinsic motivation for performance, stimulation, and knowledge. In contrast, extrinsic motivation, determined by rewards or external agents, can be classified according to the degree of internalization of behavior into: external regulation (intended to satisfy an external demand), introjected (when the behavior is directed toward self-approval or the avoidance of anxiety and guilt), identified (when the behavior is valued as personally important), and integrated (when it is fully aligned with the individual’s values and self-concept). Finally, demotivation refers to the total absence of motivation, both intrinsic and extrinsic (Ryan and Deci, 1985; Ryan and Deci, 2020).

The use of fitness trackers has transformed the way individuals manage their athletic performance. While these devices can promote motivation and self-awareness, they have also been associated with adverse psychological effects. For example, Goodyear et al. (2019) note that most users report positive benefits. However, Ragatt et al. (2018) show that intensive use of fitness trackers may be related to anxiety, body dissatisfaction, and compulsive behaviors. Furthermore, quantified feedback can influence intrinsic motivation: positive feedback could increase a sense of competence and self-determined motivation, but negative feedback or pressure to achieve high social standards could undermine motivation and lead to perfectionist and self-demanding behaviors (Deci and Ryan, 1999; Etkin, 2016; Fleer, 2024). These findings suggest that, although these devices can be useful for improving performance, they can also contribute to the emergence or intensification of perfectionist behaviors as a mechanism to avoid negative emotions such as anxiety and guilt resulting from not achieving standards. These behaviors could be maintained due to maladaptive thoughts arising from exposure to feedback and negative emotions. Despite its growing use, most studies have focused on clinical populations (McDonough et al., 2021) and its potential to increase physical activity (Brickwood et al., 2019), leaving a gap in our knowledge about its relationship with classic psychological variables in the context of sports psychology. For this reason, this study aims to explore possible differences in the psychological variables described above (perfectionism, motivation, and anxiety) according to the use or non-use of fitness trackers.

## 2 Methods

### 2.1 Design

A comparative cross-sectional study of independent samples was conducted. Non-probabilistic incidental sampling of the “snowball” type was carried out using an online form.

### 2.2 Participants

The initial sample consisted of 123 participants. Three participants were excluded for not providing basic sociodemographic information (age and gender). Therefore, the final sample used in the main analyses comprised 120 participants (*n* = 77; 64.2% men). Participants’ ages ranged from 18 to 63 years (M = 34, SD = 11.6). However, for certain subscales it was necessary to omit between four and eight participants due to random missing items that prevented the computation of the corresponding subscale scores. Consequently, the number of valid cases ranged from 112 to 116, depending on the subscale analyzed.

The sports practiced were grouped into five categories according to physiological, technical, and regular practice criteria: endurance sports (72.5%), physical conditioning sports (19.1%), martial arts (9.16%), sports with equipment (2.5%), and sports with animals (0.83%). All participants practiced one of the aforementioned sports, with triathlon (42%) and athletics (28.8%) being the most common.

Respondents reported an average of 4.74 days of training and a median of 5 days, with a standard deviation of 1.55 days. Most people train almost 5 days a week, showing a high level of commitment to physical activity.

The hours of training per week show an average of 3.57 h and a median of 4 h, with a standard deviation of 1.66 h. Participants devote between 3 and 4 h per week to training, although there are notable differences.

Finally, 84 people (70%) used fitness trackers, one of the main reasons being to record their workouts (61%) and monitor the intensity during sports practice (48.8%). The remaining 36 people (30%) did not use fitness trackers.

### 2.3 Instruments

*Ad-hoc sociodemographic questionnaire*. Sociodemographic data were studied regarding gender, age, and country, as well as the sport practiced, the hours and number of hours practiced, and the frequency of practice, days of training per week, whether they used fitness trackers and for what purpose they used it.

*Multidimensional Perfectionism Scale-2* [Frost Multidimensional Perfectionism Scale (FMPS)] [Frost et al. (1990), using the Spanish version by Carrasco et al., 2010]. The scale consists of 36 items that measure the following facets: personal standards (e.g., “It is important for me to be totally competent in everything I do in my sport”); concern about mistakes (e.g., “If I fail in competition, I feel like a failure as a person”); doubts about actions (e.g., “I rarely feel that my training prepares me for competition”); and finally, organization (e.g., “I have and follow a routine before competition”). Responses are given on a Likert scale ranging from (1) strongly disagree to (5) strongly agree. The instrument has demonstrated adequate internal consistency (*α* = 0.70). The results showed a structure of six independent factors, of which the composite reliability was adequate, except for the coach pressure factor (Pineda-Espejel et al., 2018), which was therefore eliminated, along with the parental pressure perception scale, as the population was adult. The reliability for the sample in this study was *α* = 0.73 for the personal standards scale, *α* = 0.79 for the concern about mistakes scale, *α* = 0.66 for the doubts about actions scale, and finally *α* = 0.81 for the organization scale.

*Sport Motivation Scale* (SMS) [Pelletier et al. (1995) translated into Spanish by Balaguer et al. (2007)]. In this scale, athletes are asked to answer the question “Why do you participate in your sport?” through 28 items (Balaguer et al., 2007). The responses are on a 7-point Likert scale: (1) Does not apply to me at all and (7) Applies completely to me, with the midpoint being (4) Applies somewhat to me. Internal consistency was studied using Cronbach’s alpha. The values of the subscales ranged from 0.74 to 0.83, except for the introjected regulation subscale, which showed an alpha of 0.64, and the identified regulation subscale, which showed an alpha of 0.68 (Balaguer et al., 2007). The correlations between the subscales provided evidence for the self-determination continuum and the validity of the scale construct (Balaguer et al., 2007). The reliability for the sample in the present study was *α* = 0.82 for the intrinsic motivation scale (stimulation); *α* = 0.77 for the intrinsic motivation of performance scale; 0.81 for the intrinsic motivation of knowledge scale; *α* = 0.75 for the scale identified regulation; *α* = 0.65 for the introjected regulation scale; *α* = 0.72 for the external regulation scale; and finally, *α* = 0.69 for the amotivation scale.

*Competitive Anxiety Scale* [Sport Anxiety Scale-2 (SAS-2)] (Smith et al., 2006, adapted into Spanish by Ramis et al., 2010). This questionnaire assesses the anxiety that athletes experience when facing a competitive situation. The scale has 15 items divided into three subscales: somatic anxiety, worry, and concentration disruption. Participants must evaluate the statement “Before or while I play or compete…” before answering each item (e.g., “…I feel that my body is tense”). Each item is answered on a 4-point Likert scale ranging from (1) not at all to (4) very much. It shows the internal consistency of *α* = 0.83 for the somatic anxiety scale, *α* = 0.78 for the worry scale, and *α* = 0.73 for the concentration disruption scale. The factorial structure of the adaptation faithfully replicates that obtained by Smith et al. (2006), and the construct validity indicates that the scores are related to other psychological measures (Ramis et al., 2010; Smith et al., 2006). The reliability for the study sample was *α* = 0.86 for the worry scale, *α* = 0.82 for the somatic anxiety scale, and *α* = 0.82 for the concentration disruption scale.

### 2.4 Procedure

The study was approved by the Ethics Committee of the European University of Valencia under code CIPI/22.338. The estimated duration of the questionnaire was approximately 15 min. A non-probabilistic snowball sampling method was used (Biernacki and Waldorf, 1981), in which the participants themselves shared the link with other athletes in their circle.

All participants received detailed information about the study and signed a digital informed consent form. They were guaranteed voluntary participation, anonymity, and the possibility of withdrawing from the study at any time without justification. The research was conducted in accordance with the ethical principles established in the Declaration of Helsinki (World Medical Association, 2013), always ensuring data confidentiality and respect for participants’ rights.

### 2.5 Statistical analysis

The normality of the variables was calculated using the Shapiro–Wilk test, as well as internal consistency tests (Cronbach’s alpha) to analyze the reliability of the tests. Descriptive analyses were then performed for the variables of age, gender, sport practiced, hours and days of training, and level of athletic performance. Mann–Whitney U tests were applied to observe differences between the psychological variables of the study in question according to the use or non-use of fitness trackers. In the case of analyses with missing values in any of the variables of interest, it was decided to exclude the participant from the specific analysis. The significance level was set at *p* < 0.05. The effect size of the variables was calculated. Finally, regression analyses were conducted to examine the potential association between the use of wearables and the psychological variables (perfectionism, motivation, and anxiety), while controlling age and gender.

## 3 Results

### 3.1 Mann–Whitney U test for motivation

First, the variables related to motivation showed the following results:

The comparison of the intrinsic motivation of stimulation between both groups yielded an identical median of 19.50 in both groups. The U value obtained was 1,385, with a *p* value of 0.847, which is higher than the conventional significance level (0.05). The effect size calculated using biserial rank correlation was 0.022, reflecting a negligible difference between the groups.

In the intrinsic motivation of performance variable, the group that did use fitness trackers (group 1) had a median of 18.38 compared to 19.75 in group 2. The U statistic was 1,096, with *p* = 0.157 (*p* > 0.05). The effect size was 0.169.

For the intrinsic motivation of knowledge variable, group 1 obtained a median of 17.38, while group 2 obtained 18.25. The U value was 1,140 and the *p* value was 0.113 (*p* > 0.05). The effect size was 0.186.

The median regulation identified was 15.25 for group 1 and 15.00 for group 2; U = 1,383, *p* = 0.838, and effect size 0.024.

With regard to introjected regulation, the median for group 1 was 12.88 and for group 2, 12.38. The U statistic was 1,350, *p* = 0.951 and the effect size was negative (−0.007), interpreted as indistinguishable between groups.

For external regulation, group 1 had a median of 8.25 and group 2 had a median of 9.25. U = 1,165, *p* = 0.308, and the effect size was 0.121.

Finally, in the amotivation dimension, the median was 5.25 in group 1 and 5.50 in group 2. The U statistic was 1,235, with *p* = 0.695 and small effect size (0.047).

In conclusion, the medians for each dimension were similar between the two groups. The results of the Mann–Whitney U test showed no significant differences between the groups. In no case were statistically significant differences observed, as all *p* values exceeded the threshold of 0.05. The effect sizes calculated using the biserial rank correlation were small for all variables evaluated (Tables 1–2).

**Table 1:** Mann–Whitney U test for motivation variable.

Mann–Whitney U test					
Variables	Statistic	*p*	Mean differences		Effect size
Intrinsic motivation of stimulation	1,385	0.847	−3.96e−5	Rank biserial correlation	0.022
Intrinsic motivation of performance	1,096	0.157	−1.000	Rank biserial correlation	0.169
Intrinsic motivation of knowledge	1,140	0.113	−1.000	Rank biserial correlation	0.186
Identified regulation	1,383	0.838	−0.250	Rank biserial correlation	0.024
Introjected regulation	1,350	0.951	8.29e−5	Rank biserial correlation	−0.007
External regulation	1,165	0.308	−1.000	Rank biserial correlation	0.121
Amotivation	1,235	0.695	−4.06e−6	Rank biserial correlation	0.047

H_a_ μ_1_ ≠ μ_2_.

**Table 2:** Descriptive analyses of motivation variable.

Group descriptions						
Variables	Group	*N*	Mean	Median	SD	SE
Intrinsic motivation of stimulation	1	81	18.95	19.50	3.43	0.381
	2	35	19.04	19.50	3.54	0.59
Intrinsic motivation of performance	1	80	18.28	18.38	3.36	0.375
	2	33	19.15	19.75	3.37	0.588
Intrinsic motivation of knowledge	1	80	17.11	17.38	3.84	0.429
	2	35	18.39	18.25	3.88	0.656
Identified regulation	1	81	14.72	15.25	4.36	0.484
	2	35	15.24	15.00	4.42	0.747
Introjected regulation	1	80	12.69	12.88	4.11	0.460
	2	34	12.66	12.38	5.16	0.886
External regulation	1	78	8.77	8.25	4.28	0.484
	2	34	9.90	9.25	5.14	0.881
Amotivation	1	81	6.26	5.25	3.49	0.388
	2	32	6.37	5.50	3.30	0.583

### 3.2 Mann–Whitney U test for perfectionism

Secondly, the results related to perfectionism variables show the following:

The comparison of the personal standards dimension showed a median of 2.60 in group 1 compared to 3.40 in group 2. The U value obtained was 861, with a *p*-value of 0.002, lower than the conventional significance level (0.05). The effect size calculated using biserial rank correlation was 0.369, reflecting a moderated magnitude difference between the groups.

In the variable of concern about mistakes, group 1 had a median of 1.71 compared to 2.43 in group 2. The U statistic was 873, with a statistically significant *p*-value of 0.001 (*p* < 0.05). The effect size calculated was 0.3782, indicating a moderated effect size.

For the variable doubts about actions, group 1 obtained an identical median to group 2 (2.67). The U value was 1,345 and the p value was 0.568 (*p* > 0.05). The effect size was 0.066, which shows a small effect size.

Finally, the organization variable obtained a median of 3.60 for group 1 and 3.80 for group 2; U of 1,262; a significance level of 0.460, higher than 0.05. The effect size was −0.08, indicating that the magnitude of the difference between the two groups is minimal (Tables 3–4).

**Table 3:** Mann–Whitney U test for perfectionism.

Mann–Whitney U test					
Variables	Statistic	*p*	Mean differences		Effect size
Personal standards	861	0.002	−0.600	Rank biserial correlation	0.369
Concern about mistakes	873	0.001	−0.571	Rank biserial correlation	0.378
Doubts about actions	1,345	0.568	−5.19e−5	Rank biserial correlation	0.066
Organization	1,262	0.460	0.200	Rank biserial correlation	−0.087

H_a_ μ_1_ ≠ μ_2_.

**Table 4:** Descriptive analyses for the perfectionism variable.

Group descriptions						
Variables	Group	*N*	Mean	Median	SD	SE
Personal standards	1	78	2.62	2.60	0.730	0.082
	2	35	3.23	3.40	0.953	0.161
Concern about mistakes	1	78	1.90	1.71	0.654	0.074
	2	36	2.39	2.43	0.753	0.126
Doubts about actions	1	80	2.50	2.67	0.883	0.098
	2	36	2.59	2.67	0.963	0.161
Organization	1	79	3.59	3.80	0.910	0.102
	2	35	3.49	3.60	0.849	0.143

### 3.3 Mann–Whitney U test for anxiety

In the variables related to anxiety, both somatic anxiety and worry showed no significant differences. Somatic anxiety had a median of 1.60, identical in both groups, as well as a U value of 1,372, a *p*-value of 0.763, and an insignificant effect size (0.035). Worry showed a median of 2 for group 1 and 2.20 for group 2, a U value of 1,283, and a *p*-value of 0.346, with a small effect size as well (0.109). In terms of concentration disruption, the median for group 1 was 1.20 and 1.40 for group 2; U of 1,097; a significant value of 0.034 and a small-moderate effect size (0.238) (Tables 5–6).

**Table 5:** Mann–Whitney U test for anxiety variable.

Mann–Whitney U test					
	Statistic	*p*	Mean differences		Effect size
Somatic anxiety	1,372	0.763	−4.08e−5	Rank biserial correlation	0.035
Worry	1,283	0.346	−0.200	Rank biserial correlation	0.109
Concentration disruption	1,097	0.034	−0.200	Rank biserial correlation	0.238

H_a_ μ_1_ ≠ μ_2_.

**Table 6:** Descriptive analyses for the anxiety variable.

Group descriptions						
Variables	Group	*N*	Mean	Median	SD	SE
Somatic anxiety	1	79	1.70	1.60	0.628	0.070
	2	36	1.76	1.60	0.678	0.113
Worry	1	80	2.11	2.00	0.699	0.078
	2	36	2.28	2.20	0.792	0.132
Concentration disruption	1	80	1.34	1.20	0.452	0.050
	2	36	1.52	1.40	0.505	0.084

### 3.4 Linear regression test controlling for gender and age

Controlling simultaneously for gender and age, regression analyses showed that fitness trackers use/non-use was significantly associated with personal standards, concern about mistakes, and concentration disruption.

Fitness trackers use/non-use significantly predicted personal standards: those who did not use fitness trackers scored, on average, 0.62 points higher than those who did (*β* = 0.618, *p* < 0.001), while neither age (*β* = 0.006; *p* = 0.564) nor gender (*β* = 0.156; *p* = 0.109) showed significant effects. The model had a moderate fit (*R*^2^ = 0.131).

Likewise, the use of fitness trackers significantly predicted concern about mistakes, with athletes who did not use these wearable devices reporting higher levels (*β* = 0.531, *p* < 0.001). In this model, age was negatively and significantly associated (*β* = 0.05; *p* = 0.001), while gender did not show a significant effect (*β* = 0.125; *p* = 0.220). The model explained 22.6% of the variance (*R*^2^ = 0.226).

The use/non-use of fitness trackers also significantly predicted concentration disruption: those who did not use wearables devices scored, on average, 0.20 points higher than those who did (*p* = 0.039), while age (*β* = −0.006; *p* = 0.095) and gender (*β* = −0.056; *p* = 0.537) were not significant. The model showed a modest fit (*R*^2^ = 0.065).

The rest of the regression models performed for the remaining variables did not yield significant effects. In general, the fit of the models was very low, with *R*^2^ values ranging from 0.002 to 0.122 for intrinsic motivation of stimulation (*R*^2^ = 0.002), intrinsic motivation of performance (*R*^2^ = 0.034), intrinsic motivation of knowledge (*R*^2^ = 0.029), identified regulation (*R*^2^ = 0.019), introjected regulation (*R*^2^ = 0.044), external regulation (*R*^2^ = 0.022), amotivation (*R*^2^ = 0.043), doubts about action (*R*^2^ = 0.040), organization (*R*^2^ = 0.011), and somatic anxiety (*R*^2^ = 0.061). Only the age variable was a significant predictor in two models: somatic anxiety (B = 0.005, *p* = 0.032) and worry (*R*^2^ = 0.122; B = 0.005, *p* = 0.001), showing in both cases a positive association with higher levels of anxiety. Overall, these results indicate that, except for these exceptions, the models do not show any relevant effects of the use/non-use of wearables.

## 4 Discussion

The aim of this study was to examine whether the use of fitness trackers is associated with differences in the variables of perfectionism, motivation, and anxiety among amateur athletes. In particular, we sought to determine whether there are significant differences between users and non-users in the dimensions of perfectionism (personal standards, concern about mistakes, doubts about actions, organization), motivation (intrinsic stimulation, performance knowledge; identified, introjected, external regulation; amotivation), and anxiety (somatic, worry, concentration disruption).

People who did not use fitness trackers had significantly higher levels of perfectionism, specifically in the dimensions of personal standards and concern about mistakes. No differences were found for the other factors of perfectionism. Nor were any differences found for the other variables, except for the concentration disruption variable.

First, the high personal standards detected in non-users can be explained by the tendency of certain profiles to focus on excellence by internalizing very high goals. The literature indicates that when these goals are strictly internal, they can exert such pressure that they interfere with personal resources, often responding with negative affectivity (Cooks and Ciesla, 2019; Muñoz-Villena et al., 2022; Siepmann and Kowalczuk, 2021). In this context, the non-user could be operating under a purely subjective self-assessment system. Without a monitor to provide objectivity, the perfectionist has no “ceiling” or real data to validate their effort, which can lead to a spiral of increasing demands to ensure that their standard of excellence is being met (Cooks and Ciesla, 2019; Muñoz-Villena et al., 2022; Siepmann and Kowalczuk, 2021).

Second, concern about mistakes is also significantly higher in the non-user group than in the user group. The literature suggests that individuals with a fear of failure tend to use cognitive processes such as selective attention or external attributions to protect their self-efficacy (Cooks and Ciesla, 2019; Muñoz-Villena et al., 2022). For example, a fitness tracker provides a constant source of external attribution. If the user does not achieve a goal, they can blame external factors recorded by the device (lack of time, poor sleep detected by the watch, etc.) and can also compare this with aspects of their training that did go well, which leads them to broaden their perspective and counteract the effects of selective attention.

Non-users, being more concerned about making mistakes, would lack this “external validator” of reality or comparison with positive aspects. Without objective data to quantify progress or explain poor performance, any deviation from their personal standard may be perceived as a personal failure, increasing the feelings of guilt and shame associated with the maladaptive dimensions of perfectionism.

It is paradoxical that those who are most concerned about mistakes choose to avoid the use of fitness trackers suggesting avoidant behavior. The literature offers an explanation: quantified feedback can be perceived as a threat to autonomy and competence if goals are not achieved (Etkin, 2016) and therefore, in the dynamics of reward seeking and harm avoidance (Gray, 1987), the person would opt for non-exposure. For a person with a high concern for mistakes, the possibility of a device explicitly showing them that they have not met a goal (such as 10,000 steps) can generate great pressure and fear of not complying with the norms (Busch et al., 2020). In fact, it has been observed that individuals with maladaptive perfectionist tendencies sometimes increase their performance when feedback decreases (Nuss et al., 2021). Therefore, it is possible that subjects with high perfectionism avoid using fitness trackers as a strategy to avoid negative feedback, which would drastically decrease their sense of competence and increase their anxiety.

Moreover, according to the results obtained in the linear regression model, age was negatively associated with concern over mistakes. One possible explanation is that more mature people have had more opportunities to experience a variety of situations which may contribute to the development of greater self-confidence, self-compassion, and more effective coping strategies. These resources may help them adjust their expectations about performance, relativize potential mistakes, and avoid generalizing failures (Daniilidou, 2023).

Finally, the dimension of personal standards among non-users could be linked to a search for authenticity (Russell et al., 2022). While the use of sports apps such as Strava often leads to social comparisons and pressure to present oneself to others, the perfectionist non-user may be trying to keep their standards purely linked to their identity, without the “contamination” of competition or popularity promoted by commercial devices (Busch et al., 2020). However, this lack of social support and planning tools (which devices do offer) could leave these individuals more vulnerable to distraction and internal pressure from their own unattainable standards (Mercer et al., 2016; Karapanos et al., 2016).

While these results show a link between fitness trackers use and lower perfectionism, it is essential to consider a bidirectional interpretation. It is equally plausible that athletes with lower levels of self-oriented perfectionism and less concern about mistakes are more predisposed to adopt wearable electronic devices in the first place, rather than the device itself reducing these traits.

On the other hand, regarding the motivation variable, statistical analyses did not reveal significant differences between groups. Therefore, the null hypothesis is maintained.

The absence of significant differences in motivation between the two groups is of particular interest. Although tools such as Strava or step monitors can increase motivation by satisfying the need for competition and offering social support, this relationship may be offset by other factors (Ryan and Deci, 2000; Karapanos et al., 2016; Russell et al., 2022).

The literature indicates that external feedback is a “double-edged sword”: while seeing progress increases motivation, if the activity is perceived as being externally controlled by the device, perceived autonomy decreases, which can reduce intrinsic motivation. In addition, many devices and their respective mobile applications focus on competition and popularity rather than on relationships or actual self-efficacy. This could explain why they do not generate a net increase in motivation compared to those who do not use them (Mercer et al., 2016; Karapanos et al., 2016). This idea contrasts with the results obtained by Kerner and Goodyear (2020), who observed that the use of the Fitbit physical activity monitor could make people pay more attention to physical movement and body function, thereby increasing intrinsic motivation and reducing the importance of other aspects such as body image or results. At this point, it is important to highlight the importance that motives may have in mediating emotional and cognitive responses between users and non-users (Chen et al., 2024).

About the anxiety variable, both somatic anxiety and worry showed no consistent differences between groups. However, the variable concentration disruption showed significant differences, with higher levels in those who did not use wearables electronic devices. This result shows that fitness trackers could function as a cognitive anchor: when users practice sports with fitness trackers, they can pay constant attention to information about their performance. This information could cause thoughts to be oriented toward achieving the observed goal and thinking about cognitive and physical strategies to achieve it. Without this type of observable and constant anchor, non-users may find it easier to generate other types of cognitions unrelated to the activity they are performing. Furthermore, fitness trackers could increase task-oriented cognitive load, which activates attentional control networks and suppresses the processing of irrelevant stimuli, thereby reducing distractibility (Sörqvist et al., 2016).

This is consistent with the findings of Ryan et al. (2019) and Russell et al. (2022) that fitness trackers offer a generally positive experience with few negative implications. For example, the use of performance-based goals (such as 10,000 steps) provides a clear structure that could reduce uncertainty or distraction in the task (Busch et al., 2020; Fleer, 2024). However, this idea contrasts with other studies. For example, in the systematic review conducted by Chen et al. (2024), 42 of 65 articles obtained reported both negative emotions and maladaptive cognitions (guilt, anxiety, pressure and stress, frustration, etc.). It also contrasts with the relationship evidenced between perfectionist traits and negative affect or anxiety (Xiong et al., 2024).

Although no significant differences were observed in the present study, the literature warns that, for people who are not very conscientious or physically inactive, these same external goals can generate pressure or uncomfortable feelings (Lupton, 2017). Therefore, one of the reasons why hardly any differences were observed could be due to the characteristics of the sample: amateur athletes with frequent, intense, and stable training routines for years.

The low significance found reinforces the conclusion that, although monitoring activity can generate ambiguous and sometimes unpleasant feelings, these do not appear to substantially threaten overall psychological well-being or induce elevated levels of anxiety compared to non-users.

In conclusion, although an association was found between the use of fitness trackers and lower self-directed perfectionism possibly by providing an objective, external frame of reference for success, this does not imply a causal effect. The findings may reflect a specific profile of users who are already more flexible. The stability in motivation between groups suggests that the benefits in perceived competence offered by these devices may be balanced by a loss of personal autonomy and that increases or decreases in motivation may be more associated with the reasons for use than with the use itself. Finally, quantified feedback could be functioning as a cognitive anchor that allows users to maintain a measurable and observable goal at all times, eliminating subjective thoughts and distractions.

### 4.1 Limitations and future lines of research

This study has several limitations that should be considered when interpreting the findings. The descriptive, cross-sectional design prevents causal relationships from being established between the variables analyzed. Although significant differences were observed between groups, these cannot be attributed to certainty to the use of fitness trackers. In addition, the sample size (N = 120), obtained through snowball sampling, and the marked imbalance between groups (approximately 70% users and 30% non-users) condition statistical power, hinder comparability, and condition internal validity. Online recruitment may have favored the participation of athletes with greater technological affinity, homogenizing the sample and limiting its representativeness.

Instrumental aspects must also be considered: the exclusive use of self-reports exposes them to social desirability biases, and reliability below 0.70 in some subscales (e.g., external and identified regulation) may distort the observed associations. Furthermore, the proportion of endurance athletes (72.5%) introduces a bias linked to psychological characteristics specific to these disciplines, making it difficult to generalize to other types of sports. Finally, the lack of previous evidence on the psychological effects of using fitness trackers in amateur athletes limits the possibilities for comparison and contextualization of the results.

These limitations provide clear guidelines for future research. For future studies, it is recommended to increase the sample size, use more representative sampling procedures, and combine digital and face-to-face recruitment strategies to include profiles with different levels of technological literacy. It is also recommended to work with balanced groups of users and non-users and to ensure a homogeneous distribution across sports disciplines. It is also recommended to include in new lines of research and studies objectives that improve the internal validity of conclusions with multivariate models and covariate control.

### 4.2 Implications

The results of this study have important implications for research and practice in sports psychology. First, they suggest that the use of fitness trackers may be linked to different psychological responses depending on the user’s profile and the reasons for using them. This information is useful for coaches, sports psychologists, and other professionals when assessing the potential link of these devices on athletes’ well-being.

The association between dimensions of perfectionism, extrinsic motivation, and anxiety highlights the need to pay attention to more vulnerable psychological profiles. In certain cases, intensive use of fitness trackers could reinforce dynamics of self-demand, control, or social comparison, especially in people with a strong concern for mistakes or rigid self-evaluation. This underscores the importance of promoting conscious and self-regulated use of these devices, preventing quantified feedback from becoming a source of external pressure.

Furthermore, these findings contribute to the development of an emerging line of research on the psychological effects of fitness trackers in the sports context. The evidence obtained can guide the design of personalized psychological interventions that promote more self-determined motivation and prevent possible negative effects resulting from the intensive use of these electronic devices.

Finally, there is a need for further research into the role of mediating variables such as self-efficacy, emotional regulation, and the type of feedback perceived, which could modulate the psychological fitness trackers associations.

## Data Availability

The raw data supporting the conclusions of this article will be made available by the authors, without undue reservation.
